# A Hidden Pathway to a Major Concern: The Role of Pyroptosis in Inducing Myocardial Infarction Reperfusion Injury and Emerging Therapeutic Targets

**DOI:** 10.7759/cureus.98370

**Published:** 2025-12-03

**Authors:** Zain A Mohammed, Mohamedanas Mohamedfaruk Patni, Malak M Qassim, Noor M Naji, Ibrahim K Al Abid, Ahmad Kharoufeh, Ayman A Agha, Radwan A Aloti

**Affiliations:** 1 Medicine and Surgery, RAK Medical and Health Sciences University, Ras Al Khaimah, ARE; 2 Community Medicine, RAK Medical and Health Sciences University, Ras Al Khaimah, ARE; 3 Medicine, RAK Medical and Health Sciences University, Ras Al Khaimah, ARE; 4 Orthopedics, RAK Medical and Health Sciences University, Ras Al Khaimah, ARE

**Keywords:** cardioprotection, caspase-1, clinical trials, colchicine, dapansutrile, gasdermin d, myocardial infarction, myocardial infarction reperfusion injury, nlrp3 inflammasome, pyroptosis

## Abstract

Myocardial infarction reperfusion injury (MIRI) is a paradoxical phenomenon. Restoration of blood flow to the heart potentially saves our lives, but at the same time, kills other heart cells. This impact on final infarct size counteracts the advantages of revascularization. While oxidative stress and calcium overload have long been the focus of cell demise, disease pathways that lead to cell death are classic as well, particularly the highly inflammatory pyroptosis.

This review will provide a comprehensive discussion on the established pathophysiology of MIRI and its current therapies, followed by an extensive discussion on pyrotosis. It also seeks to examine the therapeutic potential of targeting pyroptosis, evaluating drugs from preclinical development to active clinical trials.

The oxidative burst, calcium overload, mPTP (mitochondrial permeability transition pore) opening, and inflammation are all causes of the classical MIRI model. Although investigations have been undertaken for several decades, a therapeutic agent specifically for MIRI has not yet been approved. Trials of agents like cyclosporine A and remote ischemic conditioning have yielded mixed results. About pyroptosis, the pathway gets activated by signals like mtDNA and ROS, hence it triggers the NLRP3 inflammasome, leading to caspase-1 activation and gasdermin D (GSDMD) separation, forming lytic openings in the plasma membrane. It activates the release of pro-inflammatory cytokines interleukin (IL)-1β and IL-18 that further enhance tissue damage. MCC950, OLT1177, and VX-765 are NLRP3, caspase-1, and GSDMD inhibitors that decrease the infarct size in mice prior to use in humans. The clinical IL-1β antagonist canakinumab was effective in reducing recurrent events in the CANTOS (Cardiovascular Risk Reduction Study (Reduction in Recurrent Major CV Disease Events)) trial. Colchicine is beneficial in chronic coronary disease. A drug called Dapansutrile is undergoing phase II trials for new indications, and it is an NLRP3 inhibitor.

Pyroptosis is an important contributor to MIRI, linking cardiomyocyte death to potent inflammation. Focusing on this can help expand our aim to look for more potential therapeutics. The re-purposing of existing drugs, such as colchicine and disulfiram, and the development of new, specific NLRP3 inhibitors represent a promising pipeline toward finally achieving clinically relevant cardioprotection that enhances the prognosis in patients suffering from myocardial infarction.

## Introduction and background

ST-segment elevation myocardial infarction (STEMI) is the most acute manifestation of ischemic heart disease, which is the world's most common health issue [1]. Primary percutaneous coronary intervention (PCI) is the standard of care for STEMI, and timely reperfusion has been demonstrated to significantly reduce infarct size and improve survival rates [2]. Although it is a life-saving therapy, reperfusion itself can be associated with adverse consequences. Restoring blood flow can trigger a series of molecular events that lead to further and irreversible injury to the affected myocardium. This damage is called myocardial infarction- reperfusion injury (MIRI) [3].

MIRI is not a trivial component. It may contribute as much as 50% of the final infarct size. MIRI is an exciting battleground with a wealth of opportunities for therapeutic gains in cardiology [4]. MIRI may manifest as persistent myocardial dysfunction following reperfusion, microvascular obstruction, reperfusion-induced ventricular arrhythmias, or cardiomyocyte death, all of which can contribute to impaired ventricular contractility and progression to heart failure [5]. For decades, MIRI mitigation efforts have relied on a pathophysiological model that relies on a few interacting pillars. Despite the existence of evidence that many interventions could be beneficial to patients, the translation of these interventions into everyday clinical practice has been difficult. This is termed the cardioprotection translation gap [6]. Numerous interventions, such as ischemic conditioning strategies, mitochondrial permeability transition pore inhibitors (e.g., cyclosporine A), adenosine therapy, early intravenous beta-blockers, and Na+/H+ exchanger (NHE) inhibitors, have demonstrated robust cardioprotection in preclinical or early clinical studies, yet consistently failed to yield meaningful benefit in large-scale trials, exemplifying the persistent cardioprotection translation gap.

Recent years have seen a change in this paradigm, as we begin to realize that regulated cell death (RCD) is occurring outside of apoptosis. Of these, the highly inflammatory type of RCD pyroptosis has been raised as a critical participant [7]. In opposition to apoptotic cell death, where the cells are cleared in an anti‐inflammatory manner by phagocytosis, pyroptotic cells are lysed with the release of proinflammatory contents that heighten the local sterile inflammatory response and therefore worsen tissue damage [8]. This review will detail first the accepted pathophysiology of MIRI and historical clinical trials that have focused on these classical pathways. Subsequently, it will focus on the mechanism of pyroptosis as well as its selective activation in the reperfused heart and evidence from preclinical models. At the end, we will include the current data of therapeutic strategies focusing on pyroptosis, assessing the story of drugs from beginning to bedside, and discussing the future direction of this promising field.

## Review

Methods

This article was conducted as a narrative literature review with a structured but non-systematic search strategy. A targeted search of PubMed, Scopus, and Web of Science was performed from database inception to April 2025. The search used Boolean operators to combine key concepts, including terms such as “myocardial ischemia-reperfusion injury” OR “MIRI”, paired with “pyroptosis” OR “inflammasome” OR “NLRP3” OR “GSDMD”, and further linked to therapeutic terms such as “therapy”, “treatment”, “intervention”, or “cardioprotection”. An example search string was: (“myocardial ischemia reperfusion injury” OR “MIRI”) AND (pyroptosis OR inflammasome OR NLRP3 OR GSDMD) AND (therapy OR treatment OR intervention). Both recent and historically important publications were included to provide mechanistic continuity and to capture foundational developments in the field.

Eligible studies were peer-reviewed, published in English, and relevant to the mechanisms of myocardial ischemia-reperfusion injury, the molecular pathways of pyroptosis or inflammasome signaling, or therapeutic strategies targeting these mechanisms. As this work was intended as a narrative review, no Preferred Reporting Items for Systematic reviews and Meta-Analyses (PRISMA) checklist, risk-of-bias assessment, or formal study-selection diagram was employed; instead, articles were chosen based on their scientific relevance, depth of mechanistic insight, and conceptual contribution. Additional references were identified through manual screening of citations to ensure the inclusion of influential or emerging evidence.

The search initially identified 612 records. After removing 127 duplicates, 485 unique titles and abstracts were screened. A total of 412 articles were excluded for being outside the scope of cardiac tissue injury, pyroptosis pathways, or related biomedical mechanisms. The remaining 73 articles underwent full-text review, after which 25 were excluded due to insufficient mechanistic data, lack of English text, or inability to obtain the full article. Ultimately, 48 studies met the criteria and were included in the qualitative synthesis.

Classical pathophysiology of MIRI and past therapeutic endeavors

The reperfusion injury is multi-mechanistic and complex in nature. Knowledge of these classical pathways is important to place the current novelty of intervention in pyroptosis into context.

*Oxidative Burst* 

The main mechanism by which MIRI can get triggered is by the huge outburst production of reactive oxygen species (ROS) due to the sudden reperfusion to the heart cells after an ischemic period. Ischemia disturbs the mitochondrial electron transport chain; furthermore, the rapid resupply of oxygen to the system will produce significant ROS, especially superoxide anion [9], to the point where defense mechanisms like superoxide mutase and glutathione peroxidase cannot handle these changes, causing alterations to the DNA, proteins, and lipids with induction of inflammatory cascades [10].

*Calcium Overload* 

Ionic equilibrium inside cells is disturbed by ischemia. Intracellular Na+ buildup occurs when the ATP-dependent Na+/K+ pump is inhibited. Na+ levels are further raised when the Na+/H+ exchanger (NHE) is subsequently activated to rectify intracellular acidosis. This high intracellular Na+ causes a significant Ca2+ influx through the Na+/Ca2+ exchanger's (NCX) reverse mode after reperfusion [11]. Proteases (calpains), phospholipases, and endonucleases are activated, mitochondrial function is disrupted, and myofilament hypercontracture is encouraged by the ensuing cytosolic and mitochondrial calcium overload.

*Mitochondrial Permeability Transition Pore (mPTP) Opening* 

The main cause of the opening of the mPTP, a non-specific channel in the inner mitochondrial membrane, is the combination of oxidative stress and calcium overload [12]. In the end, the outer membrane rupture and necrotic cell death brought on by ATP depletion result from the opening of the mPTP, which also dissipates the mitochondrial membrane potential, uncouples oxidative phosphorylation, and induces osmotic swelling of the mitochondria [13].

*Aseptic Inflammation* 

**Figure 1:**
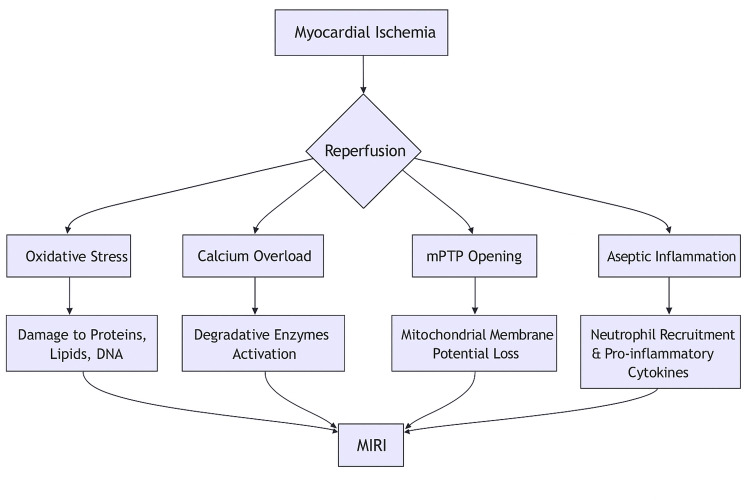
Summary of the different mechanisms that work synergistically to induce myocardial infarction reperfusion The figure was created by one of the co-authors using Microsoft Word (Microsoft Corporation, Redmond, WA, US). Acronyms: myocardial infarction reperfusion injury (MIRI), mitochondrial permeability transition pore (mPTP)

When cardiomyocytes necrotize during ischemia and reperfusion, damage-associated molecular patterns (DAMPs), such as heat shock proteins, ATP, and mitochondrial DNA, are released [14]. When pattern recognition receptors (like toll-like receptors) on nearby immune cells (like macrophages) and even cardiomyocytes detect these DAMPs, a potent innate immune response is set off. This includes the production of pro-inflammatory cytokines (including interleukin alpha (TNF-α), IL-1β, and IL-6) that further disseminate injury, as well as the recruitment and activation of neutrophils, which can block capillaries and release more dangerous enzymes and ROS [15]. The mechanism that contributes to the formation of MIRI is summarized in Figure 1.

Clinical trials targeting classical pathways

These pathophysiological findings have proven difficult to translate. The mPTP opening inhibitor cyclosporine A failed to enhance clinical outcomes in the larger CYCLONE study (Cyclosporine A in Reperfused Myocardial Infarction: Larger Outcome Trial), although showing early promise in the smaller CIRCUS trial (Cyclosporine in Reperfused Acute Myocardial Infarction Trial) [16,17]. Although it did not lower clinical event rates in the major CONDI-2/ERIC-PPCI study(Effect of Remote Ischemic Conditioning on Clinical Outcomes in Patients With Acute Myocardial Infarction / European Remote Ischemic Conditioning-Primary Percutaneous Coronary Intervention Trial), remote ischemic conditioning (RIC), a technique that involves short cycles of ischemia and reperfusion in a limb, has shown cardioprotective effects in early investigations [18]. Because exogenous antioxidants are unable to sufficiently quench the localized, explosive ROS burst at the key moment of reperfusion, they have failed everywhere [5]. The necessity for new targets and the multiplicity of harm pathways are highlighted by these failures. Table 1 highlights the clinical trials that failed to provide evidence for some therapeutic targets.

**Table 1:** Clinical trials that failed to provide evidence for some therapeutic targets Acronyms: mitochondrial permeability transition pore (mPTP), reactive oxygen species (ROS)

Trial/Intervention	Target Pathway	Key Findings/Outcome
Cyclosporine A (CYCLONE study)	mPTP opening	Failed to enhance clinical outcomes in a larger study, despite early promise in the smaller CIRCUS trial.
Remote Ischemic Conditioning (RIC) (CONDI-2/ERIC-PPCI study)	Cardioprotective effects (general)	Did not lower clinical event rates in a major study, though early investigations showed cardioprotective effects.
Exogenous Antioxidants	ROS burst	Failed to sufficiently quench localized, explosive ROS burst at reperfusion.

Pyroptosis: a primer on molecular machinery

Pyroptosis is a form of programmed cell death that depends on the Gasdermin protein family and inflammatory caspases [19].

*The Canonical Pathway* 

In MIRI, this pathway is the most well-characterized.

Priming (Signal 1): Initial inputs that activate the NF-κB pathway include DAMPs binding to toll-like receptors (e.g., TLR4). As a result, NLRP3 and the dormant cytokine precursors pro-IL-1β and pro-IL-18 are transcriptionally upregulated [20].

Activation (Signal 2): The NLRP3 inflammasome is assembled in response to a second signal that is supplied by MIRI-related insults, including lysosomal disruption, mitochondrial ROS, or K+ efflux (for example, through P2X7 receptor activation by extracellular ATP). The sensor (NLRP3), adaptor (ASC), and effector (pro-caspase-1) make up this multiprotein complex [21].

Execution: Pro-caspase-1 is autocleaved into active caspase-1 by the inflammasome. Two important substrates are then broken down by caspase-1: 1) pro-IL-1β and pro-IL-18, which are transformed into their active, secreted forms; and 2) gasdermin D (GSDMD), which releases its N-terminal domain (GSDMD-NT) from autoinhibition [22]. Large, non-selective holes are created when the GSDMD-NT oligomerizes and enters the plasma membrane. In addition to promoting the release of mature IL-1β and IL-18, this upsets osmotic balance, resulting in cell swelling and ultimately lytic death [23].

The Non-canonical Pathway

Although this pathway is less common, specially in sterile myocardial infarction re-perfusion injury, it starts when the lipopolysaccharide (LPS) inside the cytosol of the cells is recognized and sensed by human caspase 4, 5 and caspase-11 from mouse specimens; this will trigger these caspases to cleave the Gasdermin D (GSDMD) that will eventually form pores that is permeable to K+ efflux. The cleavage of the Gasdermin D triggers the pyroptotic cell death, and on top of that, K+ efflux can potentially cause secondary activation of NLRP3 inflammasomes [24].

Activation and consequences of pyroptosis in MIRI

The sterile inflammatory environment in the reperfused myocardium is full of canonical pyroptosis pathway triggers.

Inducers: Reperfusion-induced ROS burst [25], extracellular ATP from necrotic cells signaling through the P2X7 receptor [26], and mitochondrial DNA released from damaged organelles [27] are important activators.

**Figure 2:**
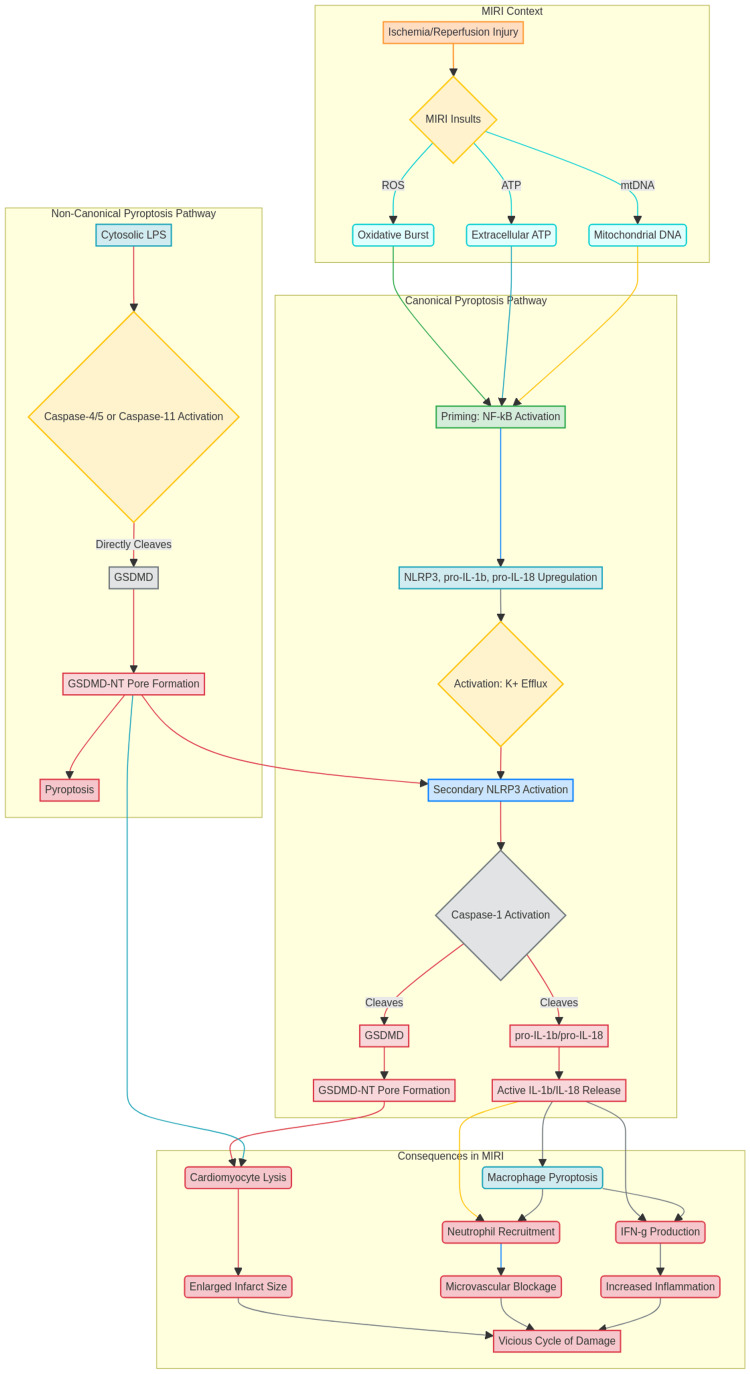
Pyroptosis pathways that lead to the induction of myocardial infarction reperfusion injury This figure was created by one of the co-authors using Microsoft Word (Microsoft Corporation, Redmond, WA, US). Abbreviations: Gasdermin D (GSDMD), myocardial infarction reperfusion injury (MIRI), NOD-like receptor protein 3 (NLRP3)

Cellular execution: Both cardiac macrophages and cardiomyocytes have been shown to exhibit pyroptosis. It is a direct cause of irreversible cell death in cardiomyocytes, which enlarges the infarct. As an "inflammatory amplifier," pyroptosis causes macrophages to release a lot of IL-1β and IL-18 [28]. While IL-18 works in concert with IL-12 to trigger the production of interferon gamma (IFN-γ), which further fuels inflammation and tissue damage, IL-1β encourages neutrophil recruitment and adherence, aggravating microvascular blockage [29]. Because of this, a vicious cycle is created in which early cell death leads to increased inflammation, which, in turn, encourages additional pyroptosis. Figure 2 compares both the canonical and non-canonical pathways in pyroptosis. 

Therapeutic targeting of pyroptosis in MIRI: from bench to bedside

The step-by-step process of pyroptosis provides several treatment avenues. The development of medications that target this route is described in the section that follows.

NLRP3 Inflammasome Inhibitors

NLRP3 is a prime target since it is the main hub for integrating danger signals.

MCC950/CRID3: A key component of preclinical research has been this strong and targeted small-molecule NLRP3 inhibitor. It prevents the construction and activation of the NLRP3 inflammasome. The administration of MCC950 at the moment of reperfusion dramatically lowers infarct size, improves heart function, and attenuates inflammation in rodent and pig MIRI models, according to numerous studies conducted between 2020 and 2024 [30,31]. Although it remains a gold-standard experimental instrument, its clinical development was apparently stopped due to possible liver toxicity, despite its substantial preclinical efficacy.

OLT1177 (Dapansutrile): In human trials for acute heart failure and gout, an oral NLRP3 inhibitor has shown a good safety profile. It successfully decreases infarct size in MIRI models, according to preclinical evidence [32]. One of the most sophisticated NLRP3 inhibitors as of 2025, it represents a direct translational avenue for this target and has active plans for phase II trials in acute myocardial infarction.

Colchicine: It has been demonstrated that this well-known anti-inflammatory medication, which is used to treat gout and pericarditis, inhibits the NLRP3 inflammasome on several levels. Its effectiveness in lowering cardiovascular events in patients with post-MI and chronic coronary syndrome, respectively, was proven by the seminal COLCOT (Colchicine Cardiovascular Outcomes Trial) and LoDoCo2 (Low-Dose Colchicine 2 Trial) trials [33,34]. The big, continuing COLCORONA (Colchicine Coronavirus SARS-CoV2) trial [35] is assessing its role in post-MI treatment, even though it hasn't been evaluated in the acute MI setting yet. Its good safety and cost profile make it a strong contender for repurposing in MIRI.

Caspase-1 Inhibitors

The maturation of cytokines and GSDMD are prevented by directly blocking the effector caspase.

VX-765 (Belnacasan): In mice models of MIRI, this oral caspase-1 inhibitor significantly reduced infarct size and pyroptosis, demonstrating cardioprotection [36]. It offers solid evidence that caspase-1 is a target, even though clinical development for other reasons was stopped.

Ac-YVAD-cmk: This cell-permeable peptide inhibitor, commonly used in experimental conditions, consistently demonstrates protective advantages against MIRI by blocking IL-1β/IL-18 production and cell death [37].

*Gasdermin D (GSDMD) Inhibitors* 

One direct method to stop lytic cell death is to block the last executor of pyroptosis.

Disulfiram: Disulfiram, an FDA-approved medication for alcohol use disorder, was unexpectedly found to be a strong and precise covalent inhibitor of GSDMD pore formation [38]. Disulfiram treatment during reperfusion provides strong protection against MIRI in mice, according to several studies conducted in 2023-2024. Because of its established safety profile for humans, it is a prime candidate for therapeutic repurposing [39].

Necrosulfonamide: Because it also targets MLKL (necroptosis executor), this drug lacks specificity even though it inhibits the pore-forming activity of GSDMD. Although dual inhibition is a drawback, its application in MIRI models has demonstrated advantages [40]. Table 2 summarizes the potential therapeutic targets in the pyroptosis pathway.

**Table 2:** Emerging potential therapeutic targets in the pyroptosis pathway Abbreviations: NOD-like receptor protein 3 (NLRP3), myocardial infarction reperfusion injury (MIRI)

Therapeutic Target	Drug/Compound	Mechanism/Notes
NLRP3 Inflammasome Inhibitors	MCC950/CRID3	Strong and targeted small-molecule NLRP3 inhibitor; prevents inflammasome assembly and activation; preclinical efficacy, but clinical development stopped due to possible liver toxicity.
	OLT1177 (Dapansutrile)	Oral NLRP3 inhibitor with good safety profile in human trials; decreases infarct size in MIRI models; active plans for phase II trials.
	Colchicine	Well-known anti-inflammatory medication; inhibits NLRP3 inflammasome at several levels; effective in lowering cardiovascular events in post-MI and chronic coronary syndrome patients.
Caspase-1 Inhibitors	VX-765 (Belnacasan)	Oral caspase-1 inhibitor; significantly reduced infarct size and pyroptosis in mice MIRI models; clinical development stopped for other reasons.
	Ac-YVAD-cmk	Cell-permeable peptide inhibitor: utilized in experiments; prevents cell death and IL-1β/IL-18 production.
Gasdermin D (GSDMD) Inhibitors	Disulfiram	FDA-approved drug; strong and precise covalent inhibitor of GSDMD pore formation; strong protection against MIRI in mice; prime candidate for therapeutic repurposing.
	Necrosulfonamide	Inhibits GSDMD pore-forming activity; also targets MLKL (necroptosis executor), thus lacks specificity.

Targeting Downstream Cytokines

It is possible to decrease damage without stopping the first cell death by blocking the inflammatory output of pyroptosis.

Anakinra: The VCU-ART (Virginia Commonwealth University - Anakinra Remodeling Trials (Anakinra to Prevent Post-infarction Remodeling)) pilot trials have evaluated the recombinant IL-1 receptor antagonist in patients with STEMI. Clinical evidence for the function of IL-1 in post-MI injury was provided by these trials, which demonstrated that Anakinra improved ventricular remodeling and decreased systemic inflammation [41].

Canakinumab: Targeting inflammation (particularly IL-1β with Canakinumab) lowers recurrent cardiovascular events in stable post-MI patients with residual inflammatory risk, according to conclusive human validation from the big CANTOS study (Cardiovascular Risk Reduction Study (Reduction in Recurrent Major CV Disease Events)) trial [42]. Targeting upstream inflammasomes like NLRP3 in acute coronary syndromes is a paradigm shift that this trial implicitly supported.

Discussion

This thorough analysis emphasizes that pyroptosis is one of the perpetrators and amplifiers of tissue damage in MIRI, not just a bystander. Its combination of lytic, pro-inflammatory cell death, caspase activation, and danger sensing produces a potent engine that propels myocardial damage after the initial ischemia assault. Its therapeutic focus is well-supported by the steady and strong cardioprotection seen with inhibitors that target each node of this pathway, from NLRP3 and caspase-1 to GSDMD, in a variety of animal models.

The present situation is really encouraging. We have a pipeline of promising medication candidates and a thorough grasp of the mechanism for the first time. Repurposing well-known medications provides a quick route to clinical impact. Colchicine is a good option for prompt investigation in acute STEMI trials because of its shown cardiovascular benefits and affordable price. With its recently identified particular effect on GSDMD, disulfiram offers yet another intriguing repurposing option with a well-established safety record. In addition to these, the creation of new, targeted medications such as dapansutrile (OLT1177) can potentially be safer and more effective treatments.

But there are still a number of difficulties and uncertainties. It is necessary to use cell-specific knockout models to further clarify the relative contributions of pyroptosis in various cell types (cardiomyocytes vs. macrophages) to the overall damage. Moreover, there is a complicated and probably context-dependent interaction between pyroptosis and other RCD processes, including ferroptosis and necroptosis [43]. These pathways, although mechanistically distinct, share upstream triggers, such as mitochondrial dysfunction, ROS accumulation, calcium overload, and DAMP release, creating a highly interconnected, regulated cell death network rather than isolated parallel pathways. For example, mitochondrial ROS and oxidized mtDNA, which activate NLRP3-driven pyroptosis, also promote lipid peroxidation and iron-dependent injury characteristic of ferroptosis. Similarly, potassium efflux induced by GSDMD pore formation can potentiate RIPK1/RIPK3/MLKL signaling and facilitate necroptotic execution. Conversely, MLKL-mediated membrane disruption in necroptosis can further amplify inflammasome activation and IL-1β maturation. These interactions suggest that inhibiting a single pathway may be insufficient in the highly redundant microenvironment of reperfused myocardium. Increasing evidence now supports the concept of ‘PANoptosis,’ a coordinated cell death program involving pyroptosis, apoptosis, and necroptosis, which may better account for the integrated response observed in MIRI models. Recognizing this interconnectedness underscores the need for combination therapeutic approaches or upstream targets capable of simultaneously modulating multiple RCD pathways. The timing of the intervention is also crucial; pre-hospital or immediate pre-PCI methods are probably required because these medications must be given at or before reperfusion.

The interpretation of the evidence presented in this review must consider the hierarchy of evidence underpinning the field. Much of the mechanistic understanding of pyroptosis in MIRI derives from in vitro experiments and small-animal in vivo models, which provide high mechanistic resolution but limited clinical generalizability. Rodent models in particular may not fully recapitulate human myocardial physiology, inflammasome activation thresholds, or comorbid conditions present in patients with STEMI. Large-animal studies and early-phase human trials remain comparatively scarce, and only a few agents, such as colchicine, anakinra, and canakinumab, have been evaluated in clinical settings. Therefore, the therapeutic promise of targeting pyroptosis is currently supported predominantly by preclinical data, and caution is required when extrapolating these findings to clinical practice. This represents a major limitation of the current evidence base and highlights the need for more robust translational and clinical studies.

Future research has to be more intelligent because of the failure of previous MIRI efforts. Trial populations may be enriched for individuals most likely to benefit from anti-pyroptotic medication if biomarkers are used to identify patients with high "inflammasonic activity" (e.g., high plasma IL-18 or GSDMD fragments) [44]. However, we are not at the point where this can be done within reperfusion time frames for PCI. For phase III trials to show definite efficacy, the incorporation of such biomarkers will be essential.

Future directions

To effectively integrate anti-pyroptotic treatments into clinical practice in the future, a number of strategic approaches will be essential. In order to enroll patients who are most likely to benefit from these targeted interventions and increase the signal of therapeutic efficacy, future clinical trials must first take a precision medicine approach by incorporating biomarkers of pyroptotic activation, such as plasma levels of cleaved GSDMD, IL-18, or specific DAMPs. Second, considering the known redundancy in cell death pathways, a major drawback of earlier monotherapies may be addressed by looking into logical combination therapies that simultaneously inhibit pyroptosis (for example, with an NLRP3 inhibitor) and a complementary pathway like ferroptosis or necroptosis. This could result in synergistic cardioprotection. Last but not least, using artificial intelligence to combine clinical imaging and multi-omics data may aid in the discovery of new MIRI endotypes characterized by inflammatory pyroptosis, opening the door to genuinely customized treatment algorithms. Whether extinguishing the pyroptotic flame will ultimately close the long-standing cardioprotection translation gap will depend on the further development of novel, targeted GSDMD inhibitors and the ongoing clinical testing of medications like dapansutrile.

## Conclusions

Mitigating MIRI has been a longstanding and challenging endeavor, and despite decades of investigation, clinically effective cardioprotective therapies remain limited. The identification of pyroptosis as an emerging contributor to ischemia-reperfusion injury has reinvigorated the field by providing a mechanistically coherent link between sterile inflammation, cardiomyocyte loss, and adverse post-infarction remodeling. Although pyroptosis-targeted interventions demonstrate strong and consistent protective effects in preclinical models, their translational validation in humans is still in its early stages. A growing therapeutic pipeline - from repurposed agents, such as colchicine and disulfiram, to novel NLRP3 inhibitors like dapansutrile - offers promising avenues for future clinical investigation. However, meaningful cardioprotection in STEMI will require rigorously designed, biomarker-enriched clinical trials that account for the complexity and redundancy of regulated cell death pathways. As research continues to unravel these interconnected mechanisms, pyroptosis remains a compelling yet incompletely validated therapeutic target whose clinical potential may be realized over the coming decade.
